# Effects of hemodialysis adequacy on chronic kidney disease complications using latent class trajectory modeling: a real-world study based on long-term observation of Kt/V

**DOI:** 10.3389/fmed.2024.1449919

**Published:** 2024-09-19

**Authors:** Sanxi Ai, Qiuyu Xu, Gang Chen, Ke Zheng, Yan Qin, Xuemei Li

**Affiliations:** Department of Nephrology, Peking Union Medical College Hospital, Chinese Academy of Medical Sciences and Peking Union Medical College, Beijing, China

**Keywords:** dialysis adequacy, CKD complications, inflammation, latent class trajectory model, Kt/V

## Abstract

**Introduction:**

Previous studies on hemodialysis adequacy primarily focused on the association between Kt/V and survival, and low Kt/V is associated with increased mortality. There is a paucity of research on the correlation between Kt/V and chronic kidney disease (CKD) complications.

**Methods:**

The retrospective study was conducted in the blood purification center of a tertiary hospital in China from July 2020 to September 2022. It aimed to analyze the association between latent Kt/V trajectory categories and CKD complications (hypertension, anemia, mineral and bone disorder) and inflammatory markers. The latent class trajectory model was established to describe the different patterns of Kt/V changes over the observation period.

**Results:**

During the 2-year study period, 93 patients on thrice-weekly hemodialysis with residual kidney function <2 mL/min were included. In the 3-class Kt/V trajectory model, 21 patients were in class 1 with a Kt/V trajectory that declined from a higher to lower levels (from >1.6 to <1.4), 59 patients were in class 2 with Kt/V consistently in a relatively low range (around 1.4), and 13 patients were in class 3 with Kt/V stabilized around 1.6. No significant difference in CKD complications or inflammation markers was observed among the three Kt/V trajectories.

**Conclusion:**

Under the premise of adequate Kt/V, neither a stable higher Kt/V nor a declined Kt/V significantly influenced CKD complications or inflammatory markers.

## Highlights

- What was known: Kt/V is the most commonly used measure of dialysis adequacy. Previous studies primarily focused on the association between Kt/V and survival.
- This study adds: The current study investigated the association between latent Kt/V trajectory categories and CKD complications and inflammatory markers. The latent class trajectory model was established to describe the different patterns of Kt/V changes over the observation period. We found no associations between latent Kt/V trajectory categories and CKD complications or inflammatory markers under the premise of adequate dialysis.
- Potential impact: To improve CKD complications and inflammation, something beyond enhancing Kt/V should be addressed, such as volume control and medications.

## 1 Introduction

The global prevalence of kidney failure was estimated to be 0.07% in the recent years (1). In 2030, the number of people needing kidney replacement therapy is estimated to be 14.5 million worldwide (2). Hemodialysis is the most commonly offered form of kidney replacement therapy, and the hemodialysis population is snowballing globally (2). Patients on hemodialysis bear significantly higher mortality and burden of disease compared to the general population (2).

Hemodialysis adequacy refers to the effective removal of retained water and uremic toxins, aiming to control various complications associated with the uremic state and to improve survival and quality of life (3, 4). Since the advent of hemodialysis, dialysis adequacy has been a focal issue among medical researchers (5). Kt/V (urea reduction ratio) is the most commonly used measure of dialysis adequacy. Efforts have consistently been made to identify the optimal dialysis adequacy for patients undergoing dialysis treatment (6, 7).

Previous studies on Kt/V have predominantly concentrated on dialysis parameters over time, primarily focusing on the relationship between dialysis adequacy and patient survival (6–8). The HEMO study, one of the most important among its categories, showed no survival benefit at Kt/V of 1.7 versus 1.3 (7). Therefore, current guidelines recommended a target single pool Kt/V (spKt/V) of 1.4 and a minimum spKt/V of 1.2 for patients on thrice-weekly hemodialysis (9). In today’s rapidly advancing medical technology landscape, the focus for dialysis patients extends beyond mere survival to encompass the quality of life and controlling complications of chronic kidney disease (CKD). However, there has been a relative scarcity of research exploring the correlation between Kt/V and CKD-related complications. Furthermore, the assessment of individual dialysis adequacy should not solely focus on the duration of a specific period but also incorporate a longer-term trend trajectory for evaluation.

Therefore, we collected Kt/V values of regular dialysis patients over an extended period to uncover the latent trajectory categories within the data and analyzed the association between these latent Kt/V trajectory categories and CKD-related complications.

## 2 Methods

### 2.1 Cohort

This retrospective cohort study was conducted at the Blood Purification Center of Peking Union Medical College Hospital (PUMCH), a tertiary hospital in China. All patients on maintenance hemodialysis thrice weekly from July 2020 to September 2022 were screened. We excluded patients with residual kidney function >2 mL/min and those on peritoneal dialysis. In Beijing, China, the first major COVID-19 outbreak occurred from November 2022 to February 2023. During the observation period from July 2020 to September 2022, none of the hemodialysis patients in our center experienced changes in their dialysis routine due to COVID-19 infection or epidemic management. The study has been approved by the institutional review board of Peking Union Medical College Hospital (K24C2372). Written consent forms were obtained from all participants or their family members.

### 2.2 Measurement of Kt/V

All patients underwent regular monthly Kt/V measurements to assess dialysis adequacy. We collected patients’ pre- and post-dialysis blood samples to obtain urea nitrogen data. Then we calculated Kt/V using the treatment time, urea distribution volume, and ultrafiltration volume based on the Daugirdas single-pool model formula: Kt/V = −ln(R - 0.008 t) + (4–3.5R) × ΔBW/BW (10), where R is the post-dialysis urea nitrogen/pre-dialysis urea nitrogen, t is the treatment time, ΔBW is the ultrafiltration volume, and BW is the post-dialysis body weight.

### 2.3 Latent class trajectory modeling of Kt/V

The latent class trajectory model is a specialized form of finite mixture modeling designed to identify latent classes of individuals following similar progressions of a determinant over time (11). Based on these Kt/V measurements, we established a latent class trajectory model to describe the different patterns of Kt/V changes in the patients over the observation period. Model selection comprehensively considers the Akaike information criterion, the Bayesian information criterion, the sample-size adjusted Bayesian information criterion, entropy, the integrated completed likelihood 1, and the integrated completed likelihood 2. By calculating and comparing these criteria values for all candidate models, the model with the smallest values tends to be selected. At the same time, model complexity and interpretability are considered to ensure that the chosen model is suitable for our data and application scenario. Several parameters and fit indices, such as proportion in each trajectory, mean posterior probability, and visual inspection of the trajectories, were also used to identify the optimal number of trajectories (12).

### 2.4 Assessment of CKD complications and inflammation

During the two-year observation period, all patients in the cohort underwent regular assessments of hypertension, anemia, CKD mineral and bone disease (CKD-MBD), and inflammatory markers. To complete the analysis, we collected the measurement data at three time points: August 2020, August 2021, and August 2022.

Blood pressure was measured before dialysis after 5 min of quiet rest. We recorded the blood pressure for each dialysis session in the corresponding month and calculated the average as the blood pressure value for that period. We used the same method to collect the patient’s weight and body mass index (BMI) values at the three time points. We collected hemoglobin, serum iron, ferritin, transferrin saturation, serum calcium, phosphorus, and parathyroid hormone levels for anemia and CKD-MBD evaluation at the three time points. We also collected C-reactive protein and systemic immune-inflammation index (SII) measured at the three time points as inflammatory markers. The SII was calculated as SII = platelet count × neutrophil count/lymphocyte count (13). If patients did not have the corresponding laboratory indicators measured at these three time points, we used the measurements from 1 month before or after. If still unavailable, the indicator was marked as missing for that time.

### 2.5 Statistical analysis

We used the lcmm package (R Foundation for Statistical Computing) to establish latent-class trajectory models for the estimation of the trajectories of Kt/V over time. After confirming the latent class trajectory model, we grouped the cohort into three classes and descriptively analyzed the patients’ measurement data at the three points (August 2020, August 2021, and August 2022). We performed Bonferroni-adjusted t-tests to compare the pairwise differences among the CKD complications (hypertension, anemia, CKD-MBD) and inflammatory markers between the three latent class trajectory model groups at the three time points. All data analyses and graphs were performed using R 4.3.1.^1^ *p* < 0.05 was considered statistically significant.

## 3 Results

### 3.1 Characteristics of patients

During the study period, 93 patients (50 males, 43 females) were included in thrice-weekly hemodialysis with residual kidney function <2 mL/min. Their mean age was 60.7 ± 12.1 years, with a mean dialysis age (duration of dialysis) of 150 ± 64.3 months.

Table 1 presented the mean blood pressure values, hemoglobin, serum calcium, phosphorus, parathyroid hormone, and inflammation markers at the three time points (August 2020, August 2021, and August 2022). Stable mean levels of blood pressure (143–148/74–80 mmHg), hemoglobin (108–109 g/L), calcium (2.3 mmol/L), and phosphorus (1.8 mmol/L) at the three time points were observed, with satisfactory ferritin (> 200 ug/L) and transferrin saturation (> 20%) levels. The mean level of parathyroid hormone was 9 times (599 pg/mL) of the upper limit (65 pg/mL) in August 2020 and was 8 times (around 530 pg/mL) of the upper limit at the remaining two time points. The mean C-reactive protein levels were significantly higher than the upper limit (3.0 mg/L) at all three time points, with mean levels of SII between 662 and 724.

**Table 1:** Measurements at three-time points in three latent classes of Kt/V trajectory.

	Total (*N* = 93)	Class 1 (*N* = 21)	Class 2 (*N* = 59)	Class 3 (*N* = 13)	*p*-value
August 2020					
Gender-male	50 (54%)	9 (43%)	34 (58%)	7 (54%)	0.715
Age	60.7 (± 12.1)	65.0 (± 11.0)	59.1 (± 12.4)	60.8 (± 11.5)	0.294
Dialysis age	150 (± 64.3)	165 (± 73.7)	145 (± 64.1)	151 (± 48.1)	0.693
Weight	62.9 (± 13.6)	59.6 (± 12.6)	65.5 (± 13.9)	56.4 (± 10.5)	0.096
BMI	22.7 (± 4.2)	21.7 (± 3.8)	23.5 (± 4.3)	21.0 (± 3.5)	0.130
Diastolic blood pressure	80.2 (± 10.1)	81.0 (± 11.8)	80.1 (± 10.0)	79.2 (± 8.3)	0.965
Systolic blood pressure	148 (± 17.3)	153 (± 20.8)	147 (± 16.3)	142 (± 13.9)	0.339
Hemoglobin	109 (± 12.5)	107 (± 11.6)	108 (± 12.9)	118 (± 8.9)	0.069
White blood cell count	6.27 (± 1.95)	5.70 (± 1.51)	6.42 (± 2.10)	6.48 (± 1.77)	0.505
Platelet count	171 (± 57.4)	179 (± 52.4)	167 (± 62.2)	171 (± 42.4)	0.877
SII	682 (± 369)	633 (± 374)	724 (± 373)	570 (± 334)	0.508
Albumin	40.2 (± 3.5)	39.6 (± 3.1)	40.3 (± 3.8)	40.7 (± 2.3)	0.837
Serum calcium	2.27 (± 0.19)	2.27 (± 0.18)	2.25 (± 0.18)	2.36 (± 0.19)	0.276
Serum phosphorus	1.81 (± 0.57)	1.71 (± 0.47)	1.82 (± 0.61)	1.91 (± 0.50)	0.804
Serum creatinine	954 (± 229)	909 (± 247)	978 (± 230)	916 (± 198)	0.617
*C-reactive protein	8.8 (± 19.1)	6.5 (± 8.90)	10.4 (± 23.2)	5.4 (± 7.07)	0.793
Serum iron	57.4 (± 23.0)	56.4 (± 22.5)	58.3 (± 22.2)	54.8 (± 28.8)	0.964
Ferritin	265 (± 216)	238 (± 194)	272 (± 216)	271 (± 257)	0.941
Transferrin saturation	22.5 (± 8.7)	23.3 (± 9.6)	22.6 (± 8.3)	21.0 (± 9.1)	0.904
Parathyroid hormone	599 (± 637)	554 (± 440)	647 (± 745)	452 (± 259)	0.772
August 2021					
Weight	63.1 (± 13.7)	60.1 (± 13.7)	65.6 (± 13.9)	56.2 (± 10.4)	0.094
BMI	22.8 (± 4.2)	21.8 (± 4.2)	23.6 (± 4.2)	20.9 (± 3.4)	0.121
Diastolic blood pressure	76.0 (± 10.2)	76.9 (± 8.2)	75.8 (± 10.5)	75.2 (± 12.4)	0.964
Systolic blood pressure	143 (± 15.9)	146 (± 12.7)	142 (± 16.2)	138 (± 19.2)	0.566
Hemoglobin	109 (± 13.6)	111 (± 11.7)	107 (± 14.9)	111 (± 8.7)	0.589
White blood cell count	6.05 (± 2.38)	5.49 (± 1.47)	6.19 (± 2.72)	6.31 (± 1.81)	0.684
Platelet count	168 (± 53.5)	169 (± 50.0)	165 (± 56.9)	179 (± 44.3)	0.865
SII	724 (± 523)	631 (± 382)	760 (± 565)	714 (± 538)	0.814
Albumin	40.0 (± 2.5)	39.9 (± 2.59)	40.0 (± 2.41)	40.2 (± 2.85)	0.994
Serum calcium	2.27 (± 0.21)	2.29 (± 0.25)	2.24 (± 0.19)	2.34 (± 0.22)	0.386
Serum phosphorus	1.83 (± 0.59)	1.88 (± 0.63)	1.88 (± 0.60)	1.52 (± 0.37)	0.242
Serum creatinine	958 (± 249)	955 (± 237)	981 (± 259)	858 (± 215)	0.46
**C-reactive protein	7.0 (± 11.2)	5.3 (± 8.27)	8.2 (± 12.9)	4.3 (± 5.22)	0.596
**Serum iron	60.9 (± 27.1)	59.6 (± 19.6)	63.2 (± 30.3)	51.3 (± 19.0)	0.577
**Ferritin	240 (± 187)	218 (± 164)	246 (± 194)	252 (± 200)	0.945
**Transferrin saturation	24.4 (± 12.6)	24.4 (± 9.2)	25.0 (± 14.2)	21.5 (± 9.1)	0.860
***Parathyroid hormone	536 (± 549)	431 (± 254)	599 (± 670)	438 (± 176)	0.604
August 2022					
Weight	62.9 (± 13.7)	60.2 (± 13.3)	65.4 (± 13.9)	56.1 (± 11.0)	0.113
BMI	22.8 (± 4.2)	21.9 (± 4.0)	23.5 (± 4.2)	20.9 (± 3.7)	0.154
Diastolic blood pressure	74.3 (± 10.8)	76.0 (± 11.3)	72.8 (± 11.1)	78.4 (± 7.0)	0.318
Systolic blood pressure	144 (± 19.1)	147 (± 17.8)	141 (± 20.1)	148 (± 15.6)	0.486
Hemoglobin	108 (± 15.5)	107 (± 15.8)	107 (± 15.7)	110 (± 15.1)	0.964
White blood cell count	5.62 (± 1.88)	5.23 (± 1.87)	5.70 (± 1.91)	5.89 (± 1.78)	0.738
Platelet count	166 (± 60.5)	167 (± 62.9)	164 (± 60.3)	173 (± 62.1)	0.973
SII	662 (± 423)	595 (± 445)	681 (± 430)	685 (± 366)	0.880
Albumin	39.9 (± 3.4)	39.0 (± 4.0)	40.3 (± 3.4)	40.0 (± 2.4)	0.525
Serum calcium	2.28 (± 0.20)	2.29 (± 0.21)	2.27 (± 0.21)	2.35 (± 0.16)	0.623
Serum phosphorus	1.79 (± 0.56)	1.81 (± 0.53)	1.78 (± 0.60)	1.82 (± 0.47)	0.992
Serum creatinine	949 (± 231)	925 (± 251)	969 (± 238)	897 (± 151)	0.719
****C-reactive protein	9.7 (± 31.4)	19.4 (± 57.8)	6.5 (± 15.9)	6.6 (± 8.3)	0.446
*****Serum iron	60.4 (± 24.7)	58.0 (± 23.9)	63.0 (± 25.7)	53.1 (± 21.0)	0.589
*****Ferritin	203 (± 188)	208 (± 244)	197 (± 172)	220 (± 171)	0.980
*****Transferrin saturation	23.0 (± 11.1)	23.6 (± 13.2)	23.2 (± 11.0)	21.3 (± 8.54)	0.948
^#^Parathyroid hormone	527 (± 467)	463 (± 267)	543 (± 536)	553 (± 337)	0.940

Unit of measurements: age (years), dialysis age (months), weight (kg), BMI (kg/m^2^), diastolic and systolic blood pressure (mmHg), hemoglobin (g/L), white blood cell count and platelet count (10^9^/L), albumin (g/L), serum calcium and phosphorus(mmol/L), serum creatinine (μmol/L), C-reactive protein (mg/dL), serum iron (μmol/L), ferritin (μg/L), transferrin saturation (%), parathyroid hormone (pg/mL). Dialysis age represented the duration of dialysis. *Missing values for 9 patients; **missing values for 2 patients; ***missing values for 6 patients; ****missing values for 8 patients; *****missing values for 5 patients; #missing values for 15 patients; SII: Systemic immune inflammation index.

### 3.2 Latent class trajectory modeling of Kt/V

Over the two-year observation period, patients in the cohort had a median of 12 (range from 9 to 24) measurements of Kt/V. We tested latent class trajectory models from 2 to 5 classes, and the 3-class model showed the optimal performance in Akaike information criterion, Bayesian information criterion, entropy, and integrated completed likelihood. Moreover, it also exhibited balanced class proportions and better visual inspection of the trajectories (Supplementary Table S1). The posterior probability of the 3-class latent class trajectory model is shown in Supplementary Table S2.

**Figure 1:**
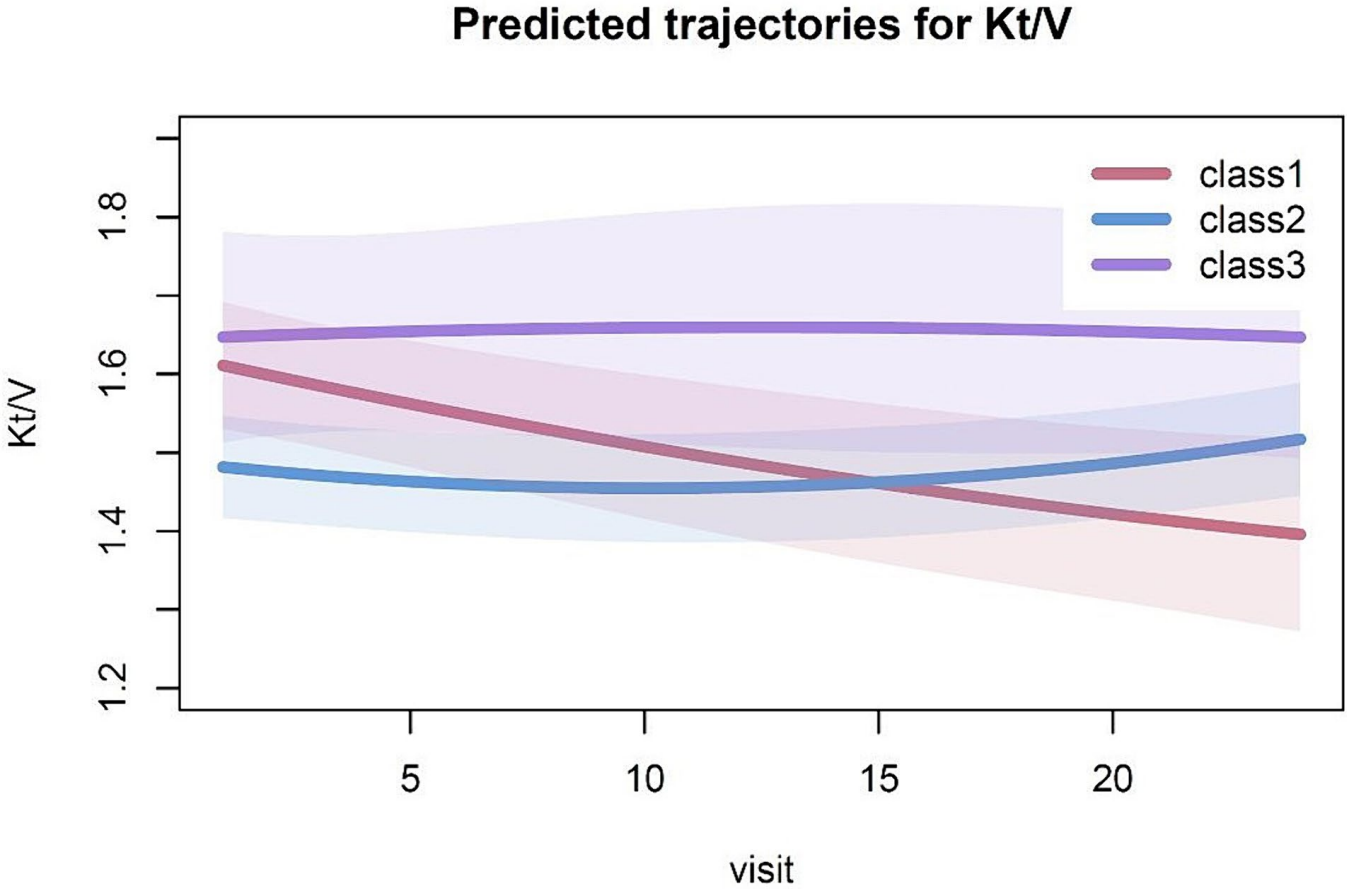
Predicted trajectories for Kt/V. The solid line indicates the estimated Kt/V trajectory for the category, while the shaded areas represent the 95% confidence intervals.

In the 3-class model, 21 patients were in class 1 with a Kt/V trajectory that declined from a higher to lower levels (from >1.6 to <1.4), and 59 patients were in class 2 with Kt/V consistently in a relatively low range (around 1.4). Another 13 patients were in class 3 with Kt/V consistently maintained in a relatively high range (around 1.6) (Figure 1).

### 3.3 Effect of Kt/V trajectory on CKD complications and inflammation

**Figure 2:**
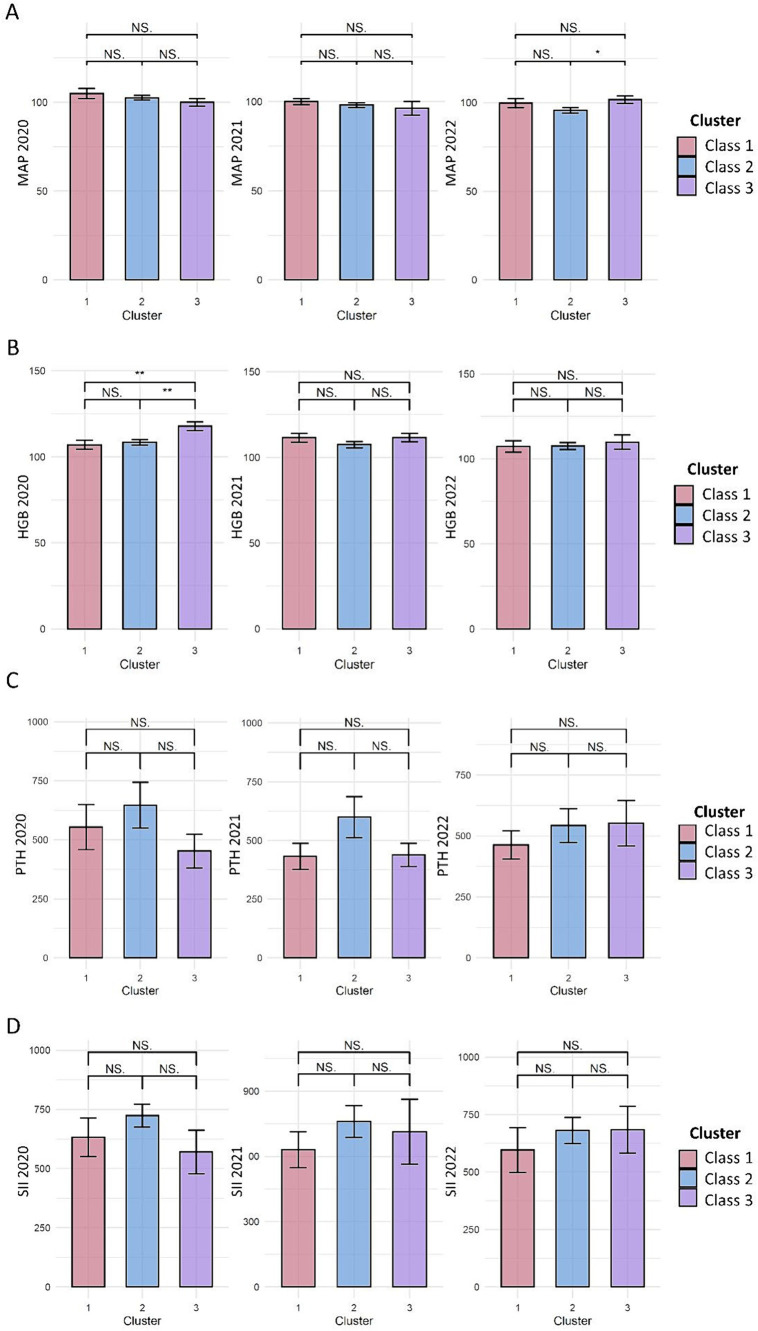
Effect of latent Kt/V trajectory classes on CKD complications and inflammation. **(A–D)** represents the comparison of mean artery pressure (MAP, mmHg), hemoglobin (HGB, g/L), parathyroid hormone (PTH, pg/ml), and systemic immune inflammation index (SII) among the three Kt/V trajectory classes, respectively. The bars indicate the mean ± standard error, with pairwise comparisons conducted to assess the differences between the three classes. NS, indicates that the comparison shows no statistically significant difference. *indicates *p* < 0.05; **indicates *p* < 0.01.

We compared the blood pressure and laboratory measurements at the three time points among the three classes of Kt/V trajectories. At the three time points, no significant differences in mean artery pressure were detected among the three classes of Kt/V trajectories (Figure 2A).

In August 2020, a higher mean hemoglobin level was observed in Kt/V trajectory 3 than in Kt/V trajectory 1 and 2, but statistical significance was not reached (*p* = 0.069). In August 2021 and August 2022, no significant difference in the hemoglobin levels was observed among the three Kt/V trajectories (Figure 2B).

At all three time points, no significant difference in parathyroid hormone level was detected among the three classes of Kt/V trajectories (Figure 2C).

Regarding inflammatory markers, the mean levels of SII and C-reactive proteins were not significantly different among the three KT/V trajectories at all three time points (Figure 2D).

## 4 Discussion

Previous studies on hemodialysis adequacy primarily focused on the association between Kt/V and survival among hemodialysis patients. They showed that low Kt/V is associated with higher mortality (6, 8), while increasing Kt/V to 1.7 showed no survival benefit compared to Kt/V of 1.3 (7). Current guidelines recommend a minimum spKt/V of 1.2 (9). The current study differed from previous studies because we focused on the association between Kt/V and CKD complications. In addition, hemodialysis patients need continuous evaluations of Kt/V and CKD complications based on long-time observations. Another strength of the current study was that we described the different patterns of Kt/V changes over 2 years using the latent class trajectory modeling. We found that, even over a long period, neither a stable higher Kt/V nor a declined Kt/V significantly influenced CKD complications or inflammatory markers under adequate dialysis.

Renal anemia is a common complication in hemodialysis patients, and erythropoiesis-stimulating agents (ESAs) are the primary treatment. Many factors may modify the response to ESAs, including iron stores, inflammatory status, dialysis adequacy, and hyperparathyroidism (14). An inverse correlation between dialysis adequacy (Kt/V) and ESA response has been demonstrated (14–17), but no correlation was observed when Kt/V is above 1.33 (14, 16). Consistent with previous findings, we observed no significant difference in hemoglobin levels in patients with higher Kt/V (1.6) compared to patients with lower Kt/V (1.4), suggesting that no additional benefit on anemia with Kt/V higher than 1.4.

Hypertension is observed in >80% of patients on maintenance hemodialysis (18). Multiple factors contribute to hypertension in dialysis patients. Among these factors, the most proximal cause is excessive intravascular volume. Previous studies have demonstrated enhanced volume removal during dialysis improved blood pressure control in patients on maintenance hemodialysis (19). Few studies have examined the association between Kt/V and blood pressure in the hemodialysis population. In the current study, we observed no correlation between Kt/V and hypertension among patients on thrice-weekly hemodialysis, consistent with previous research findings (20). This result is reasonable since hypertension is closely associated with volume removal rather than small molecular weight solute removal represented by urea Kt/V.

CKD-MBD is another common complication in people with advanced CKD. Hyperphosphatemia is central to hormonal dysregulation in CKD-MBD, and current clinical practice guidelines have recommended normalizing serum phosphorus (21). Dietary restriction and phosphate binders are the primary interventions to manage hyperphosphatemia, but with limited efficacy. A previous study suggested that the use of two dialyzers in parallel led to increased phosphate clearance and lower pre-dialysis serum phosphate in overweight patients (> 80 kg) with inadequate Kt/V (22). However, a more extensive study did not confirm this finding in hemodialysis patients with a mean Kt/V of 1.44 (23). The current study explored the correlation between Kt/V and serum phosphate, calcium, and PTH levels. We assumed that intensifying small-solute clearance by enhancing Kt/V might lead to better phosphate clearance and control of parathyroid hormone levels control. However, we found no significant differences in serum phosphate, calcium, or parathyroid hormone levels among patients with different Kt/V classes. Several reasons might explain the results. Firstly, Kt/V urea does not reflect phosphorus clearance because the phosphorus removal kinetics during dialysis differ from urea’s (24). The primary determinant of phosphorus removal is weekly dialysis time (24). Secondly, the serum phosphorus depends not only on elimination during dialysis but also on dietary intake and phosphate binders. In the current study, we did not quantify phosphate binder doses or estimate dietary phosphate intake, which might have confounded our results.

Uremic toxin retention may trigger the production of pro-inflammatory cytokines (25). A previous study showed that patients with inadequate dialysis (Kt/V < 1.2) exhibited higher levels of circulating levels of inflammatory mediators compared to patients with adequate dialysis (26). An inverse correlation between Kt/V and serum C-reactive protein level was observed previously (27). The current study found no significant difference in inflammation markers among the three Kt/V categories. Considering that almost all patients in our study had adequate dialysis, we could not determine the effect of inadequate dialysis on inflammatory markers.

The study has several limitations. Firstly, the sample size was relatively limited, which may restrain the study’s statistical power to detect subgroup differences. Secondly, we did not quantify medications for CKD complications, which may confound the results. Finally, the study was conducted in a center where most patients had adequate dialysis. We could not determine the effect of inadequate dialysis on CKD complications and inflammatory markers.

## 5 Conclusion

In conclusion, we found no associations between latent Kt/V trajectory classes and CKD complications or inflammatory markers under the premise of adequate dialysis measured by Kt/V among patients on maintenance hemodialysis thrice-weekly. Something beyond enhancing Kt/V should be addressed to improve CKD complications and inflammation, such as volume control, dietary restriction, and medications.

## Data Availability

The original contributions presented in the study are included in the article/Supplementary material, further inquiries can be directed to the corresponding author/s.

## Footnotes

^1^http://www.r-project.org
