# Supplementary material for: Effects of hemodialysis adequacy on chronic kidney disease complications using latent class trajectory modeling: a real-world study based on long-term observation of Kt/V
Source: Front Med (Lausanne). 2024 Sep 19;11:1449919. doi: 10.3389/fmed.2024.1449919 (PMC11446740; doi:10.3389/fmed.2024.1449919)
Supplement: Supplementary file 1 [file Table_1.docx]

**Table S1.** Comparison of relative fit indices for model selection

| **Number of classes** | **AIC** | **BIC** | **SABIC** | **Entropy** | **ICL1** | **ICL2** |
| --- | --- | --- | --- | --- | --- | --- |
| 2 | -1463.688 | -1425.699 | -1473.051 | 0.6142919 | -1400.835 | -1400.959 |
| 3 | -1457.589 | -1457.589 | -1469.448 | 0.5450742 | -1362.989 | -1365.537 |
| 4 | -1455.000 | -1455.000 | -1469.356 | 0.7056931 | -1358.806 | -1358.806 |
| 5 | -1453.098 | -1453.098 | -1469.951 | 0.7838043 | -1352.359 | -1353.510 |

Abbreviations: AIC: Akaike Information Criterion; BIC: Bayesian Information Criterion; SABIC: Sample-size-adjusted Bayesian Information Criterion; ICL: Integrated Classification Log-likelihood criterion computed in two ways, ICL1 = BIC - sum[pi_ig*log(pi_ig)], or ICL2 = BIC - 2*sum(log(max(pi_ig)), where the max is taken over the classes for each subject.

**Table S2.** The posterior probability of the 3-class latent class trajectory model.

|  | Probability 1 | Probability 2 | Probability 3 |
| --- | --- | --- | --- |
| Class 1 | 0.7542 | 0.1274 | 0.1184 |
| Class 2 | 0.0498 | 0.8580 | 0.0922 |
| Class 3 | 0.1352 | 0.1988 | 0.6660 |
